# Highly Pathogenic H5N1 Influenza Viruses Carry Virulence Determinants beyond the Polybasic Hemagglutinin Cleavage Site

**DOI:** 10.1371/journal.pone.0011826

**Published:** 2010-07-27

**Authors:** Jessica Bogs, Jutta Veits, Sandra Gohrbandt, Jana Hundt, Olga Stech, Angele Breithaupt, Jens P. Teifke, Thomas C. Mettenleiter, Jürgen Stech

**Affiliations:** 1 Institute of Molecular Biology, Friedrich-Loeffler-Institut, Federal Research Institute for Animal Health, Greifswald-Insel Riems, Germany; 2 Institute of Infectology, Friedrich-Loeffler-Institut, Federal Research Institute for Animal Health, Greifswald-Insel Riems, Germany; Tsinghua University, China

## Abstract

Highly pathogenic avian influenza viruses (HPAIV) originate from avirulent precursors but differ from all other influenza viruses by the presence of a polybasic cleavage site in their hemagglutinins (HA) of subtype H5 or H7. In this study, we investigated the ability of a low-pathogenic avian H5N1 strain to transform into an HPAIV. Using reverse genetics, we replaced the monobasic HA cleavage site of the low-pathogenic strain A/Teal/Germany/Wv632/2005 (H5N1) (TG05) by a polybasic motif from an HPAIV (TG05_poly_). To elucidate the virulence potential of all viral genes of HPAIV, we generated two reassortants carrying the HA from the HPAIV A/Swan/Germany/R65/06 (H5N1) (R65) plus the remaining genes from TG05 (TG05-HA_R65_) or in reversed composition the mutated TG05 HA plus the R65 genes (R65-HA_TG05poly_). In vitro, TG05_poly_ and both reassortants were able to replicate without the addition of trypsin, which is characteristic for HPAIV. Moreover, in contrast to avirulent TG05, the variants TG05_poly_, TG05-HA_R65_, and R65-HA_TG05poly_ are pathogenic in chicken to an increasing degree. Whereas the HA cleavage site mutant TG05_poly_ led to temporary non-lethal disease in all animals, the reassortant TG05-HA_R65_ caused death in 3 of 10 animals. Furthermore, the reassortant R65-HA_TG05poly_ displayed the highest lethality as 8 of 10 chickens died, resembling “natural” HPAIV strains. Taken together, acquisition of a polybasic HA cleavage site is only one necessary step for evolution of low-pathogenic H5N1 strains into HPAIV. However, these low-pathogenic strains may already have cryptic virulence potential. Moreover, besides the polybasic cleavage site, the additional virulence determinants of H5N1 HPAIV are located within the HA itself and in other viral proteins.

## Introduction

Highly pathogenic avian influenza viruses (HPAIV) are the causative agents of fowl plague [1], [2] which causes devastating economic losses in gallinaceous poultry. In addition, several HPAIV strains are able to infect humans and, therefore, are considered as potential precursors for future influenza pandemics [3]. For infection, the envelope glycoprotein hemagglutinin (HA) precursor HA0 requires proteolytic cleavage by cellular proteases into the two subunits HA1 and HA2. Mammalian and low-pathogenic avian influenza A viruses (LPAIV) carry an HA cleavage site with a monobasic motif susceptible to trypsin-like proteases which confine viral replication to the respiratory or gastrointestinal tract. In contrast, HPAIV possess a polybasic HA cleavage site cleavable by furin [4], [5], which is ubiquitous and thus supports systemic viral replication. This polybasic HA cleavage site is the prime virulence determinant of HPAIV [6], [7], [8] which originate from LPAIV precursors [4], [9], [10], [11], [12], [13], [14], [15], [16]. Acquisition of a furin recognition motif was shown to result from different events such as recombination of the HA gene with 28S ribosomal RNA [17] or with sequences encoding other viral proteins like the nucleoprotein (NP) gene of an unrelated virus [15] or the HA and matrix protein genes (M) from the same virus [16]. An alternative proposed mechanism is polymerase slippage on template regions with stable secondary structures [13], [14]. In mammalian influenza viruses, virulence determinants have been attributed to the HA [18], [19], [20], [21], [22], NA [18], [23], NS1 [24], [25], [26], [27], [28], NP and polymerase proteins [18], [29], [30], [31], [32], [33], [34]. In HPAIV, beside the polybasic HA cleavage site, the caspase cleavage motif in the M2 protein and deletions within the NA stalk region were associated with increased virulence [35], [36], [37], [38], [39], [40]. Furthermore, introduction of the NS gene from an H5N1 HPAIV into an H7N1 fowl plague strain rendered it virulent for mice [41]. Recently, we demonstrated that the acquisition of a polybasic cleavage site by an LPAIV H3N8 strain is not sufficient for immediate transformation into an HPAIV, and that additional virulence determinants other than the polybasic HA cleavage site are required [42]. However, it remained to be analyzed whether H5 or H7 LPAIV, which are considered HPAIV precursors, have to undergo further evolutionary changes prior to or after acquisition of a polybasic cleavage site. Therefore, we addressed in this study the question whether a polybasic cleavage site engineered into the HA of an H5N1 LPAIV leads to immediate transformation into an HPAIV. To elucidate the virulence potential of all viral genes of H5N1 HPAIV in chicken further, we generated two H5N1 reassortants carrying an HPAIV HA plus the remaining LPAIV genes, or, in reversed composition, the LPAIV HA with engineered polybasic cleavage site plus the HPAIV genes.

## Results

### Generation of Recombinant Viruses

As parental strains we used a recent H5N1 LPAIV isolated in Germany in 2005, A/Teal/Germany/Wv632/2005 (H5N1) [43] as well as the first HPAIV H5N1 isolate derived from the outbreak in wild swans on the island of Rügen in February 2006, A/Swan/Germany/R65/06 (H5N1) [44]. First, plasmid-based reverse genetics systems were established for both strains, resulting in the recombinant viruses TG05 (this study) and R65 [45], respectively. To introduce a polybasic HA cleavage site into TG05 (H5N1), we replaced its monobasic HA cleavage site with a polybasic motif from HPAIV A/Duck/Shanghai/13/01 (H5N1) by site-directed mutagenesis and used this plasmid for generation of the mutant TG05_poly_. Considering possible structural constraints within the HA of the parental TG05, we also adapted the amino acids adjacent to the cleavage site to those from the HA of A/Duck/Shanghai/13/01 (H5N1) (Table 1). Furthermore, we rescued two reassortants carrying the HA from HPAIV R65 plus the remaining seven genes from TG05 (TG05-HA_R65_), or, in reversed composition, the mutated TG05 HA plus the remaining R65 genes (R65-HA_TG05poly_). Remarkably, we could not rescue a mutant of TG05 with the polybasic cleavage site region from R65 suggesting structural impairments of the mutated HA.

**Table 1:** Generated viruses and their HA cleavage sites.

Abbreviation	Description	HA Cleavage Site
TG05	A/Teal/Germany/Wv632/05 (H5N1) recombinant, monobasic cleavage site	GPRNVPQKET - - - - R/G
TG05_poly_	Recombinant TG05 with polybasic cleavage site from A/Duck/Shanghai/13/01 (H5N1)	GLRNTPQRERRRKKR/G
TG05-HA_R65_	Reassortant from TG05 with HA gene from A/Swan/Germany/R65/06 (H5N1)	GLRNSPQGERRRKKR/G
R65-HA_TG05poly_	Reassortant from R65 with HA gene from TG05_poly_	GLRNTPQRERRRKKR/G

### TG05_poly_ has in-vitro properties of an HPAIV

**Figure 1:**
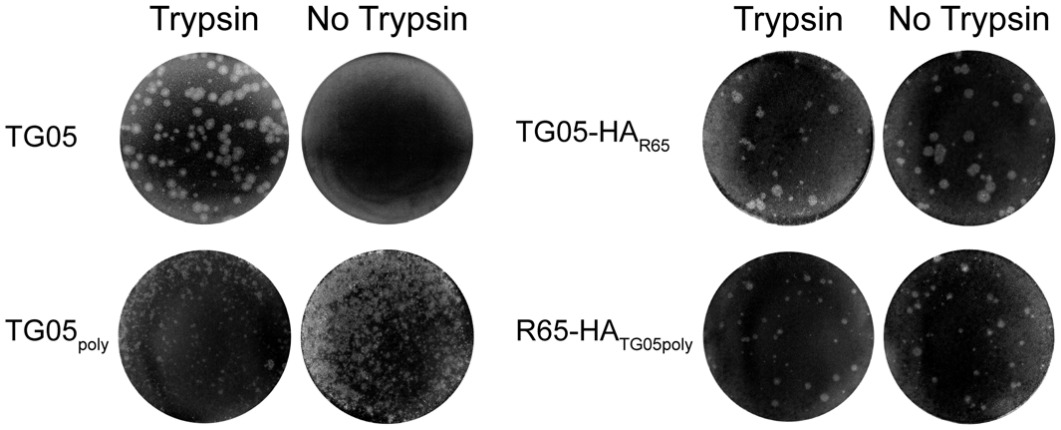
Multicycle replication in-vitro. Plaque assays of TG05 in comparison with TG05_poly_, TG05-HA_R65_, and R65-HA_TG05poly_ on MDCK cells in the presence and in the absence of trypsin.

**Figure 2:**
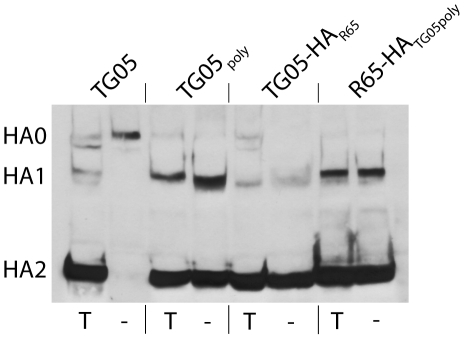
Proteolytic HA activation. Western blots of DF-1 cell lysates infected with TG05, TG05_poly_, TG05-HA_R65_ or R65-HA_TG05poly_ at multiplicity of infection of 0.1 incubated in the presence (T) and in the absence (-) of trypsin.

**Figure 3:**
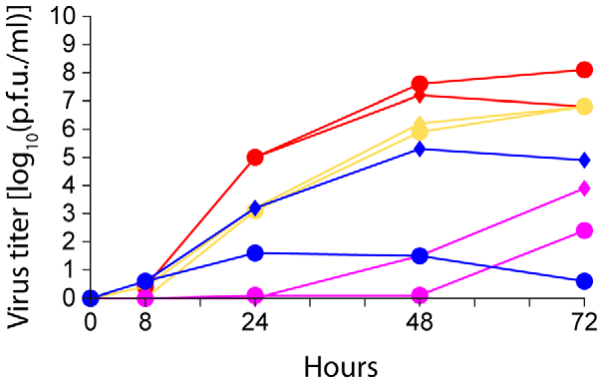
Growth kinetics. DF-1 cells were inoculated with TG05 (blue), TG05_poly_ (magenta), TG05-HA_R65_ (yellow), and R65-HA_TG05poly_ (red) at multiplicity of infection 10^−3^ in the presence (diamonds) and in the absence (circles) of trypsin.

To address the question whether the HA cleavage site mutant and the two reassortants are dependent on trypsin for multicycle replication, we performed plaque assays. Whereas the parental TG05 required exogenous trypsin for plaque formation, all the viruses with a polybasic HA cleavage site, TG05_poly_, TG05-HA_R65_, and R65-HA_TG05poly_, yielded plaques independent of trypsin (Fig. 1). Proteolytic HA cleavage was analyzed by immunoblotting. Corresponding to plaque assays, the HA0 precursor of TG05 remained uncleaved in the absence of trypsin in contrast to HA0 of TG05_poly_, TG05-HA_R65_, and R65-HA_TG05poly_ (Fig. 2), which were cleaved into the HA1 and HA2 fragments. The HA0 cleavage was incomplete in all viruses studied. Multicycle growth kinetics of TG05 showed a strong dependence on exogenous trypsin as the virus failed to replicate efficiently in the absence of trypsin. TG05_poly_ replicated both in the presence and in the absence of trypsin, although with a delay compared with the other viruses. However, both reassortant viruses TG05-HA_R65_ and R65-HA_TG05poly_ replicated to high titers irrespective of trypsin (Fig. 3). In summary, these results demonstrate that TG05_poly_, TG05-HA_R65_, and R65-HA_TG05poly_ undergo multicycle replication in the absence of trypsin in contrast to their parent virus TG05 and, thus, display HPAIV phenotypes in-vitro.

### Pathogenicity in chicken

**Figure 4:**
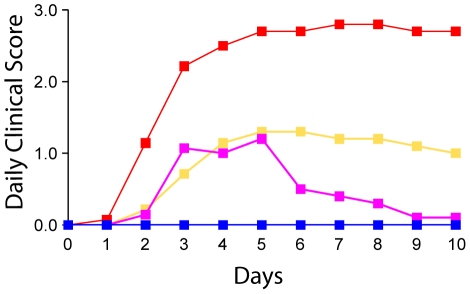
Virulence in chicken. Daily clinical score after oculonasal inoculation with 10^5^ pfu of TG05 (blue), TG05_poly_ (magenta), TG05-HA_R65_ (yellow) or R65-HA_TG05poly_ (red). The birds were observed for 10 days for clinical signs and classified as healthy (0), ill (1), severely ill (2), or dead (3); the daily clinical score was calculated from the sum of individual clinical scores from all birds divided by the number of animals per group (10 chickens).

To investigate the virulence of TG05, TG05_poly_, TG05-HA_R65_, and R65-HA_TG05poly_ in chicken, 10 animals each were infected with 10^5^ pfu of the respective virus oculonasally, and observed daily for clinical symptoms for 10 days (Table 2 and Fig. 4). In contrast to the low-pathogenic parental virus TG05 which proved to be completely innocuous as expected, the HA cleavage site mutant TG05_poly_ and the two reassortants TG05-HA_R65_ and R65-HA_TG05poly_ were pathogenic in chicken to an increasing degree. Infection with TG05_poly_ led to temporary non-lethal disease in all animals (predominantly mild symptoms such as ruffled feathers, depression or diarrhea mainly from day 3 to 6). After inoculation with TG05-HA_R65_ 3 of 10 animals died, whereas R65-HA_TG05poly_ displayed the highest lethality as 8 of 10 infected chickens died, thus exhibiting the phenotype of a natural HPAIV (Table 2).

**Table 2:** Virulence in chickens.

Virus	Clinical Score	Morbidity	Mortality
TG05	0.0	0/10	0/10
TG05_poly_	0.5	10/10	0/10
TG05-HA_R65_	0.9	8/10	3/10
R65-HA_TG05poly_	2.1	10/10	8/10

Clinical score, morbidity, and mortality after 10 days observation following oculonasal infection.

### Organ tropism and tissue lesions in chicken

On day 4 post inoculation, samples from cerebellum, cerebrum, lung, nose, trachea, caecum, duodenum, kidney, and pancreas from three (TG05, TG05_poly_ and TG05-HA_R65_) and four (R65-HA_TG05poly_) additionally infected birds per group were removed and investigated for viral spread in organs and the extent of lesions in affected tissues. No influenza virus antigen or histomorphological lesions were found in organs of TG05 infected birds. In one of the three TG05_poly_-inoculated birds, single positive neurons, pneumocytes II, endothelial cells, lymphocytes, and macrophages could be detected in cerebrum, lung, nasal conchae, trachea, caecum, and duodenum. Furthermore, in one TG05-HA_R65_-infected bird, small clusters of infected cells were present in cerebellum and cerebrum suggesting that already the introduction of the R65 HA facilitates neurotropism.

**Figure 5:**
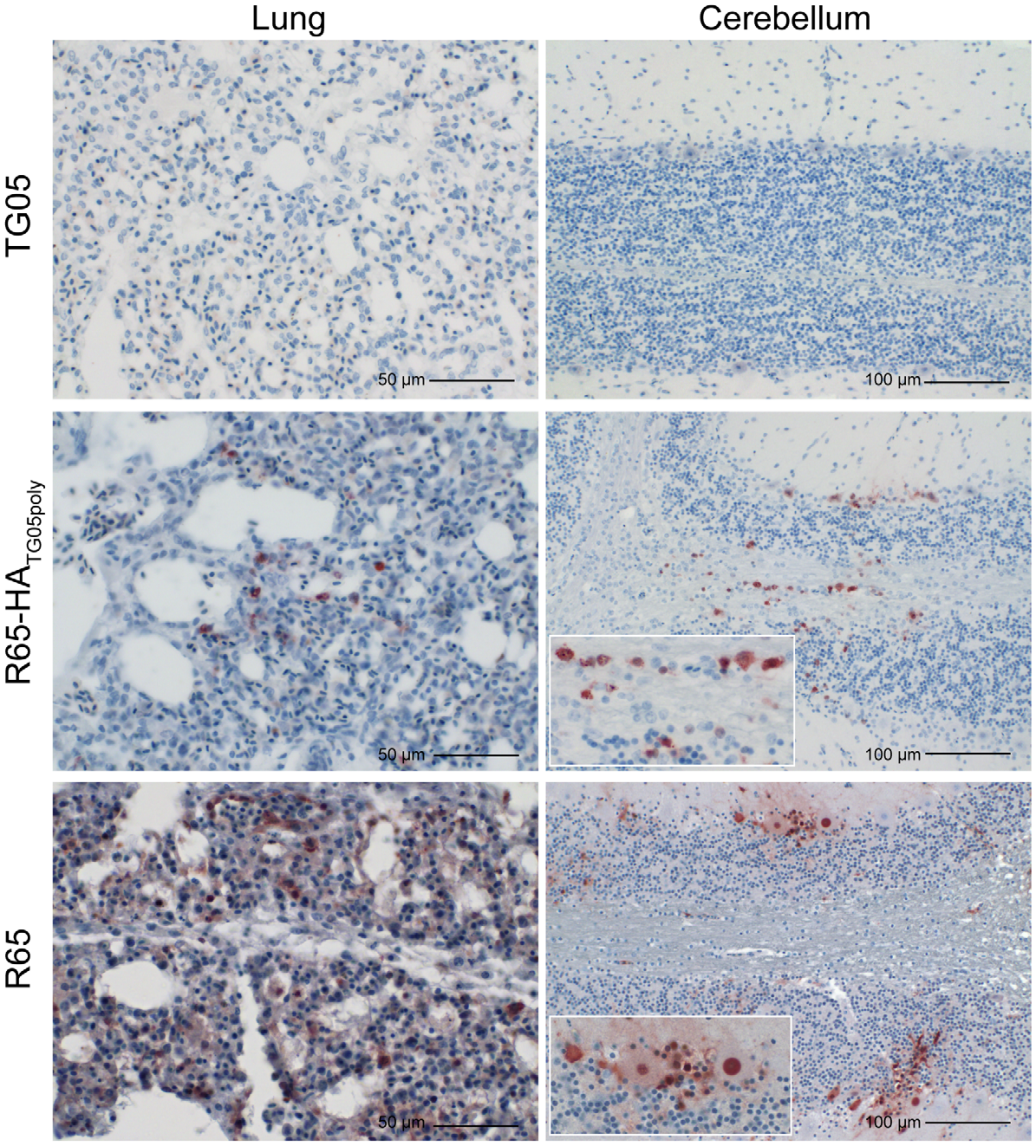
Viral organ tropism in chicken. Immunohistochemical detection of influenza A virus nucleoprotein (brown) in lung and cerebellum from chickens sacrificed on day 4 post inoculation with 10^5^ pfu of TG05 or R65-HA_TG05poly_, and from moribund chickens sacrificed on day 2 post inoculation with 10^6^ TCID_50_ R65.

All four R65-HA_TG05poly_-infected birds exhibited larger and more intensely stained clusters of infected cells within cerebellum, cerebrum, and nasal mucosae; several birds displayed single positive cells in lung, trachea, caecum, duodenum, kidney, and pancreas. After infection with the recombinant HPAIV R65 [45], the picture of infected cells appeared more intense (Table 3, Fig. 5). These results demonstrate a broader organ tropism of R65-HA_TG05poly_-inoculated birds compared with TG05_poly_-inoculated birds demonstrating the relevance of the other viral genes for systemic spread.

**Table 3:** Organ tropism and tissue lesions on day 4.

4 dpi		Cerebellum	Cerebrum	Lung	Nose	Trachea	Caecum	Duodenum	Kidney	Pancreas
TG05	IHC	−/−/−	−/−/−	−/−/−	−/−/−	−/−/−	−/−/−	−/−/−	−/−/−	−/−/−
	IHC positive cell types									
	HE histo-pathology	none	none	none	lymphohistiocytic rhinitis	none	None	none	none	none
TG05_poly_	IHC	−/−/−	**+**/−/−	**+**/−/−	**+**/−/−	**+**/−/−	**+**/−/−	**+**/−/−	−/−/−	−/−/−
	IHC positive cell types		neurons, glial cells	lymphocytes, pneumocytes II	endothelium, macrophages	endothelium, macrophages, lymphocytes	serosal endothelium, lymphocytes, macrophages	serosal endothelium, lymphocytes, macrophages		
	HE histo-pathology	none	neuropil vacuolisation (mild edema), glial cell proliferation	edema, focal parabronchial necrosis	mixed cellular rhinitis; comb: skin necrosis, dermatitis, epidermal necrosis	mixed cellular tracheitis	serositis	serositis	none	none
TG05-HA_R65_	IHC	**++**/−/−	**++**/−/−	−/−/−	**++**/−/−	−/−/−	−/−/−	−/−/−	−/−/−	−/−/−
	IHC positive cell types	neurons, glial cells, ependymal cells	neurons, glial cells, ependymal cells		glandular epithelium, lymphocytes, macrophages					
	HE histo-pathology	neuronal degeneration	lymphocytic encephalitis, neuronal degeneration, glial cell proliferation	mild edema	glandular epithelial degeneration, lymphohistiocytic rhinitis; comb: dermatitis	none	none	none	none	parenchymal degeneration
R65-HA_TG05poly_	IHC	**++**/**++**/**++**/**++**	**++**/**++**/**++**/**++**	−/**+**/**++**/+	**++**/**+++**/**++**/**++**	**+**/**++**/−/**+**	−/−/**+**/**+**	−/**+**/**+**/−	−/**+**/−/−	−/**+**/−/**++**
	IHC positive cell types	neurons, glial cells, ependymal cells	neurons, glial cells, ependymal cells	macrophages, lymphocytes, pneumocytes II, endothelium	epithelial cells (glands, skin, feather follicle, nasal conchae); glial cells (N. trigeminus), lymphocytes, macrophages,	endothelium, muscle cells, lymphocytes, macrophages	endothelium, lymphocytes, macrophages	lymphocytes, macrophages	tubular epithelium, macrophages, lymphocytes	macrophages, acinar cells
	HE histo-pathology	neuronal degeneration	neuronal necrosis, neuronal degeneration, glial cell proliferation, multifocal	mild edema; BALT: lymphocyte necrosis and heterophil infiltration, interstitial pneumonia	mixed cellular necrotizing rhinitis with epithelial proliferation and hemorrhage, blood vessel necrosis, serocellular crusts, dermatitis	lymphohistio-cytic tracheitis	GALT: lymphocyte necrosis with heterophil infiltration	none	none	acinar necrosis, mixed cellular pancreatitis

Immunohistochemical detection (IHC) of influenza virus nucleoprotein antigen and HE staining of organs from chickens after intranasal inoculation.

On day 10 post inoculation, organs were removed from four surviving birds each of the TG05, TG05_poly_ and TG05-HA_R65_-infected groups. No antigen was detectable in tissues of these birds, except for a few positive neurons and glial cells in the cerebellum from one TG05-HA_R65_-infected bird, and in the cerebrum from two surviving R65-HA_TG05poly_-infected animals, as well as inflammatory cells in the nasal mucosa from one of those birds, demonstrating the recovery of the animals (Table S1).

Taken together, the presence of influenza virus antigen corresponded to degeneration and necrosis of the affected tissues accompanied by lymphocytic and histiocytic infiltration. Extent of organ tropism and severity of lesions corroborate with the course of disease. In HPAIV, viral neurotropism can be facilitated by the HA gene solely and systemic spread is mediated also by the internal protein genes.

## Discussion

Influenza virus virulence is a polygenic trait which had been established by generating reassortants neurovirulent for mice from two apathogenic strains, and reassortants apathogenic in chicken from two virulent strains [46], [47]. Furthermore, reassortment studies based on two H5N1 HPAIV strains revealed that the exchange of the HA, NP, M2 or NS1 proteins resulted in increased mortalities and expanded tissue tropism in chicken. Moreover, replacement of the PB2 or PB1 proteins led to decreased replication in tissues and, consequently, a decrease in virulence [48]. These findings can be attributed to involvement of these proteins in virulence or to constraints of gene segment exchanges by reassortment in HPAIV. However, the prime determinant of virulence is the polybasic HA cleavage site [6], [7], [8] as its conversion into a monobasic motif invariably resulted in loss of virulence [7]. On the other hand, introduction of such a polybasic motif into the HA cleavage site of a low-pathogenic H3N8 strain did not lead to transformation into an HPAIV indicating the existence of additional virulence determinants in the HA and/or the other viral proteins [42].

To shed more light on virulence determinants required for evolution of HPAIV which, in nature, derive from low-pathogenic precursors of subtypes H5 or H7 [4], [9], [10], [11], [12], [13], [14], [15], [16], we addressed the question whether a low-pathogenic avian strain of the H5N1 subtype would transform into a highly pathogenic strain after introduction of a polybasic HA cleavage site. For that purpose, we replaced the monobasic HA cleavage site from TG05 by a polybasic motif from the HPAIV A/Duck/Shanghai/13/01 (H5N1) resulting in the mutant TG05_poly_. Remarkably, attempts to obtain a similar mutant with the HA cleavage site of HPAIV R65 were not successful indicating structural incompatibilities with the TG05 HA.

Although TG05_poly_ was able to replicate in the absence of trypsin in cell-culture, and, thus, exhibits the in-vitro phenotype of an HPAIV, this mutant caused only temporary mild disease in chicken and, accordingly, could be detected only in isolated single cells accompanied with minor lesions in affected organs. These observations differ from those obtained by the H3N8 polybasic cleavage site mutants generated from the LPAIV A/Duck/Ukraine/1/1963 which caused considerably less symptoms in chicken [42] compared with TG05_poly_ indicating that the low-pathogenic H5N1 strain already carried cryptic virulence determinants. Correspondingly, repeated air sac inoculation of a low-pathogenic H5N3 isolate in 1-day-old-chickens did not lead to high virulence for 4-to-6-week-old chickens, whereas only two further passages in brain yielded an HPAIV [49].

To demonstrate the existence of virulence determinants besides the polybasic cleavage site in the HA or in other genes of H5N1 HPAIV, we generated two reassortants, TG05-HA_R65_ carrying the HA from the HPAIV R65 plus the remaining seven genes from TG05 and, in reverse gene constellation, R65-HA_TG05poly_ composed of the mutated TG05 HA plus the other R65 genes. Whereas TG05-HA_R65_ already had a lethality of 30%, R65-HA_TG05poly_ caused a lethality of 80%, displaying the phenotype of a “natural” HPAIV. These findings indicate that in H5N1 HPAIV the HA gene alone provides for a certain level of virulence, whereas the complete viral genetic background influences pathogenicity to a major extent.

Compared with the very broad organ tropism of the HPAIV R65 in chicken [50], the mutant TG05_poly_ and the two reassortants TG05-HA_R65_ and R65-HA_TG05poly_ displayed generally less viral spread in organs and less extensive lesions in surrounding tissues. However, spread and lesions increased with the two reassortants from which R65-HA_TG05poly_ exhibited the phenotype of an authentic HPAIV. This finding again emphasizes the relevance of HA and the other viral genes for virulence. Moreover, all birds which had viral antigen detectable in inner organs, i.e. those infected by TG05_poly_, TG05-HA_R65_ or R65-HA_TG05poly_ also exhibited a neuronal infection. Thus, the presence of the HPAIV R65 HA alone in a LPAIV background suffices for viral neurotropism which is considered pivotal for the fatal course of fowl plague [51], [52], [53].

Previous studies on HPAIV strains revealed that (1) removal of the polybasic HA cleavage site results in a drastic decrease in pathogenicity [7]; (2) virulence is a multigenic trait as reassortants derived from HPAI strains can be attenuated [46], two LPAI strains can reassort to a highly virulent virus [47] and reassortants with exchanged HA, NP, M or NS, PB2 or PB1 gene have an altered virulence [48]; (3) NA stalk deletion leads to a certain increase in virulence in an LPAIV [40]; and (4) NS1 can increase virulence of an HPAIV further [41]. Most of these studies started from HPAIV and measured a decline in pathogenicity, which demonstrates that the identified alterations were required for full expression of virulence, or resulted from reassortment events which are prone to incompatibilities. No study so far addressed the question whether acquisition of a polybasic cleavage site is sufficient to transform a LPAIV H5 precursor into a highly pathogenic H5 virus. However, knowledge on this problem is most relevant, since it is required for development of a science-based assessment of the risk of HPAIV emergence from LPAIV field strains. Therefore, we introduced a polybasic HA cleavage site into the low-pathogenic H5N1 strain TG05, a potential HPAI precursor virus, in order to model the early evolution of HPAIV. This approach complements the current knowledge on the relative importance of virulence determinants and HPAIV evolution from the opposite point of view.

Taken together, acquisition of a polybasic HA cleavage site is only one necessary step for evolution of H5N1 HPAIV from low-pathogenic precursor strains, which may already have cryptic virulence potential. Moreover, besides the polybasic HA cleavage site, additional virulence determinants of HPAIV are located within the HA itself and in the other viral proteins. Knowledge of these virulence factors is crucial for an assessment of the risk of transformation of any LPAIV to HPAIV which is highly relevant for the control of notifiable LPAIV H5 or H7 infections in poultry.

## Material and Methods

### Ethics Statement

The animal experiments were evaluated by the responsible ethics committee of the State Office for Agriculture, Food Safety and Fishery in Mecklenburg-Western Pomerania (LALFF M-V) and gained governmental approval (registration number LALLF M-V/TSD/7221.3-1.1-018/07).

### Cells and Viruses

Human embryonic kidney 293T [32] and Madin-Darby canine kidney (MDCK) cells [32] were maintained in DMEM containing 10% fetal calf serum (FCS). Chicken fibroblast cells (DF-1) were cultured in ISCOVES DMEM containing 10% FCS in the absence of antibiotics. The low-pathogenic avian influenza A virus A/Teal/Germany/Wv632/2005 (H5N1) (TG05) was isolated from a healthy green winged teal *(Anas crecca)* [43]. The sequences of cloned viral genes have been submitted to Genbank (accession numbers CY061882-9). A/Swan/Germany/R65/06 (H5N1) (R65) was isolated from a dead swan during the HPAIV outbreak in Rügen, Germany, 2006 (Genbank accession numbers DQ464354-DQ464361) [44]. Both native viruses were propagated in 11-day-old embryonated chicken eggs.

### Generation of recombinant viruses

Viral genes from TG05 were cloned into the plasmid pHWSccdB as described in [45]. Site-directed mutagenesis of the HA cleavage site region was performed by the Quikchange™ protocol (primer sequences available upon request). All recombinant viruses were rescued essentially as described [32] with the addition of plasmids expressing the polymerase proteins and the nucleoprotein genes from A/PR/8/34 (H1N1) (a kind gift from Peter Palese) except for R65-HA_TG05poly_. The recombinant R65 [45] differs from the native isolate [44] by 4 nucleotide exchanges in the PB2 gene (DQ464357): C1009T corresponding to the amino acid replacement L329F, in the PB1 gene (DQ464361): A1603G and G1604T corresponding to the amino acid replacement S527V, and in the HA gene (DQ464354): C1737T in non-coding region. TG05 was propagated in embryonated eggs, all other recombinant viruses were grown on MDCK cells. Gene composition and HA cleavage site of the generated viruses were verified by sequencing of amplicons from reverse transcription-PCR (data not shown). All viruses with polybasic HA cleavage site were handled under BSL3+ conditions.

### Plaque assay and growth curves

Plaque assays were performed on MDCK cells as described previously [54] with 400 µl inoculum either in the presence of 2 µg/ml N-tosyl-L-phenylalanine chloromethyl ketone (TPCK)-treated trypsin (Sigma, Taufkirchen, Germany), or in the absence of trypsin. For determination of growth curves, DF-1 cells were inoculated at a multiplicity of infection of 10^−3^ in the presence of 1 µg/ml TPCK-treated trypsin or in the absence of any exogenous protease. From two independent experiments, supernatants were harvested at 0, 8, 24, 48, and 72 h post inoculation and resulting infectious virus was titrated by plaque assay on MDCK cells in the presence of trypsin.

### Western blots

DF-1 cells were infected at a multiplicity of infection of 0.1 in the presence of either 1.0 µg/ml TPCK-treated trypsin or no exogenous protease in ISCOVES DMEM and 0.2% bovine serum albumin (BSA) (MP Medicals, Heidelberg) until a cytopathic effect appeared (48 h for TG05, TG05-HA_R65_, and R65-HA_TG05poly_; 7 d for TG05_poly_). Cells were lysed with 4× Laemmli buffer [55] containing 4% sodium dodecyl sulfate (SDS) and were inactivated by heating at 95°C for 5 min. Lysates were separated on a 10%-SDS polyacrylamid gel and electrotransferred to a nitrocellulose membrane. For detection of HA, a polyclonal rabbit antibody to the HA protein from A/Chicken/Vietnam/P41/2005 (H5N1), expressed by a vaccinia virus vector [56], (1∶20,000; incubated 1 h at room temperature) and as secondary antibody a rabbit-specific goat immunoglobulin G fragment conjugated with horseradish peroxidase (1∶10,000; 1 h at room temperature, Biovision, USA) were used. Antibody binding was visualized by chemiluminescence (Supersignal West Pico Chemiluminescence Kit from Pierce, Bonn).

### Animal experiments

Ten 2-week-old White Leghorn specific-pathogen-free chickens per group were infected oculonasally with 10^5^ PFU per animal. Each bird was observed daily for 10 days for clinical signs and classified as healthy (0), ill (1) (exhibiting one of the following: respiratory symptoms, depression, diarrhea, cyanosis, edema, or central nervous symptoms), severely ill (2) (severe or more than one of the previously mentioned symptoms), or dead (3) as described previously [57]. When birds are too sick to eat or drink, they are killed humanely and scored as dead to the next observation day [57].

### Histopathology and immunohistochemistry

Samples from cerebellum, cerebrum, lung, nose, trachea, caecum, duodenum, kidney, and pancreas of additionally infected chicken were taken on day 4 and 10 post infection, formalin-fixed and processed for paraffin-wax-embedding according to standardized procedures. Immunohistochemical detection of influenza A virus nucleoprotein (NP) and hematoxylin eosin staining was performed as described [58].

For R65 [45], samples from cerebellum and lung were taken on day 2 from two 3-week old chickens infected oculonasally with 10^6^ TCID_50_/animal.

## Supporting Information

Table S1 Organ tropism and tissue lesions on day 10. Immunohistochemical detection (IHC) of influenza virus nucleoprotein antigen and HE staining of organs from chickens after intranasal inoculation.
(0.04 MB DOC)
