# Supplementary material for: Highly Pathogenic H5N1 Influenza Viruses Carry Virulence Determinants beyond the Polybasic Hemagglutinin Cleavage Site
Source: PLoS One. 2010 Jul 27;5(7):e11826. doi: 10.1371/journal.pone.0011826 (PMC2910732; doi:10.1371/journal.pone.0011826)
Supplement: Table S1 — Organ tropism and tissue lesions on day 10. Immunohistochemical detection (IHC) of influenza virus nucleoprotein antigen and HE staining of organs from chickens after intranasal inoculation. (0.04 MB DOC) [file pone.0011826.s001.doc]

| 10 dpi |  | Cerebellum | Cerebrum | Lung | Nose | Trachea | Caecum | Duodenum | Kidney | Pancreas |
| --- | --- | --- | --- | --- | --- | --- | --- | --- | --- | --- |
| TG05 | IHC | -/-/-/- | -/-/-/- | -/-/-/- | -/-/-/- | -/-/-/- | -/-/-/- | -/-/-/- | -/-/-/- | -/-/-/- |
|  | IHC positive cell types |  |  |  |  |  |  |  |  |  |
|  | HE histo-pathology | none | none | none | lymphohistiocytic rhinitis with hemorrhage | none | none | none | none | none |
| TG05_poly_ | IHC | -/-/-/- | -/-/-/- | -/-/-/- | -/-/-/- | -/-/-/- | -/-/-/- | -/-/-/- | -/-/-/- | -/-/-/- |
|  | IHC positive cell types |  |  |  |  |  |  |  |  |  |
|  | HE histo-pathology | none | none | none | lymphohistiocytic rhinitis, epithelial desquamation with cellular debris, epithelial hyperplasy;  comb: lymphocytic dermatitis | none | none | none | none | none |
| TG05-HA_R65_ | IHC | -/-/-/- | -/**+**/-/- | -/-/-/- | -/-/-/- | -/-/-/- | -/-/-/- | -/-/-/- | -/-/-/- | -/-/-/- |
|  | IHC positive cell types |  | multifocal single neurons, glial cells |  |  |  |  |  |  |  |
|  | HE histo-pathology | lymphocytic meningoencephalitis, neuronal degeneration, glial cell proliferation | lymphocytic meningoencephalitis, neuronal degeneration, glial cell proliferation | none | lymphocytic rhinitis;  comb and skin:  lymphocytic dermatitis | none | none | none | none | lymphohistiocytic serositis |
| R65-HA_TG05poly_ | IHC | -/- | **(+)**/**(+)** | -/- | -/**(+)** | -/- | -/- | -/- | -/- | -/- |
|  | IHC positive cell types |  | neurons, glial cells |  | lymphocytes, macrophages |  |  |  |  |  |
|  | HE histo-pathology | none | lymphocytic meningoencephalitis, neuron degeneration, glial cell proliferation | none | lymphocytic rhinitis | none | none | none | none | none |
